# Multi-modal few-shot learning for anthesis prediction of individual wheat plants

**DOI:** 10.1016/j.plaphe.2025.100091

**Published:** 2025-07-21

**Authors:** Yiting Xie, Stuart J. Roy, Rhiannon K. Schilling, Huajian Liu

**Affiliations:** a University of Adelaide, Australia; b Australian Plant Phenomics Network, Australia; c ARC Training Centre for Future Crops Development, Australia; d South Australian Research & Development Institute, Australia; e Flinders University, Australia

**Keywords:** Plant phenomics, Anthesis prediction, Multimodal approach, Few-shot learning, Individual wheat phenotyping

## Abstract

Anthesis prediction is crucial for breeding wheat. While current tools provide estimates of average anthesis at the field scale, they fail to address the needs of breeders who require accurate predictions for individual plants. Hybrid breeders have to finalize their plans for pollination at least 10 days before such flowering is due and biotechnology field trials in the United States and Australia must report to regulators 7–14 days before the first plant flowers. Currently, predicting anthesis of individual wheat plants is a labour-intensive, inefficient, and costly process. Individual wheat of the same cultivar within the same field may exhibit substantial variations in anthesis timing, due to significant variations in their immediate surroundings. In this study, we developed an efficient and cost-effective machine vision approach to predict anthesis of individual wheat plants. By integrating RGB imagery with in-situ meteorological data, our multimodal framework simplifies the anthesis prediction problem into binary or three-class classification tasks, aligning with breeders' requirements in individual wheat flowering prediction on the crucial days before anthesis. Furthermore, we incorporated a few-shot learning method to improve the model's adaptability across different growth environments and to address the challenge of limited training data. The model achieved an F1 score above 0.8 in all planting settings.

## 1 Introduction

Wheat (*Triticum aestivum*) is one of the most important cereal crops, playing a vital role in food security. Predicting key phenological stages, such as anthesis, is critical for wheat breeding and agronomic management strategies. Accurate prediction enables the selection of high-yield, climate-resilient varieties. Current research on prediction of wheat anthesis relies on genetic, environmental, and simulation-based computer models. Multi-locus genotype-based models use genetic markers to simulate anthesis dates, achieving a root mean square error of 2.3 days [1]. Environmental models, which emphasize the effects of temperature and photoperiod through growing degree days (GDD) and photoperiod algorithms to predict phenology, have demonstrated a strong link between plant flowering time, temperature, and day length, as highlighted by Ishaque et al. [2]. Adjusting daily thermal time accumulation with soil water content has further improved anthesis prediction accuracy in wheat [3]. The Agricultural Production Systems sIMulator (APSIM), a wheat simulation model, integrates temperature, photoperiod, and vernalization requirements, achieving 72 ​% accuracy in predicting phenological development in the Australian wheat belt area [4,5]. These studies have significantly improved our ability to predict time to anthesis in a group of wheat plants in large-scale planting areas. However, the predictions required by breeders and researchers are often more complex and precise requiring prediction times for individual wheat plants.

In wheat breeding and certain aspects of research, the precise prediction of anthesis date for individual wheat plants is critical. In hybrid wheat breeding, accurate anthesis predictions are crucial for timing manual cross-pollination interventions, which must occur two to five days before the actual anthesis of predominantly self-pollinating wheat [6,7]. Advance knowledge of the anthesis schedule, ideally eight to ten days ahead, is essential for effective planning and coordination of pollination activities, particularly in field settings where manual verification of optimal pollination timing is labour-intensive [8]. Precise scheduling is vital for achieving high success rates in pollination, crucial for optimal seed quality and yield. Managing the limited number of plants per hybrid variety in breeding programs requires meticulous timing and management to successfully execute pollination strategies. Notably, the variability in flowering times within even the same field trial can range from five to ten days, reflecting not only macro-environmental influences like temperature, sunlight, and rainfall but also micro-environmental factors such as variable soil conditions including soil texture, nutrient, and water retention capacity [9,10]. Furthermore, the prediction of flowering time of individual wheat plants is critical for breeders and researchers using biotechnology. The United States Animal & Plant Health Inspection Service (APHIS) requires that researchers conducting plant biotechnology field trials must regularly inspect the plants to predict the flowering time for individual plants. Flowering time must be accurately predicted no later than 7 calendar days before the first anticipated flowering date of the field trial for isolation of volunteer plants [11,12]. This timeframe ensures sufficient time for spatial isolation or the removal of flowering buds to prevent biosafety issues or to terminate the trial if necessary. In Australia, the Office of the Gene Technology Regulator (OGTR) requires similar stringent regulations, mandating accurate forecasting of the initial anthesis event for individual plants at least 14 days in advance of anthesis, to mitigate risks associated with genetically modified (GM) plant field trials [13]. This is to mitigate the risks associated with genetically modified (GM) plant field trials and the spread of pollen to sexually compatible weed/crop species. Prediction of anthesis 14 days in advance allows alterations in management practices of the area surrounding GM field trials to remove sexually compatible species that are near flowering [14,15]. To comply with these OGTR regulations, GM field trials, require labour-intensive, frequent inspections by highly trained personnel who visit GM trial sites at least bi-weekly to monitor and document growth stages to ensure accurate anthesis predictions [13]. This process is costly and time-consuming, necessitating a dedicated team. Automatic determination of flowering time for individual plants, using computer vision and machine learning, would be beneficial to improve compliance and reduce costs. Currently, most automatic flowering time predictions focus on entire fields rather than individual plants. Due to variation in anthesis dates among wheat plants within the same field, there is a need for more efficient, automated methods to predict individual flowering times, reducing reliance on manual inspections and potentially lowering costs and labour for regulatory compliance.

High-throughput plant phenotyping, which uses advanced imaging and analysis techniques to measure a wide range of plant traits quickly and accurately, has become transformative in agricultural research and plant breeding [16,17]. In wheat breeding, it has successfully detected growth stages, monitored leaf structures, and developed automated ear counting methods [[18], [19], [20]]. However, these sensor-based image studies have to date focused only on the static state of wheat. The time to anthesis in wheat can be influenced by environmental factors, such as rainfall, minimum and maximum temperature and duration of sunlight [10,21], making it difficult to accurately predict flowering time based on a single static state. Consequently, relying solely on image-based phenotyping is insufficient for addressing dynamic issues influenced by environmental conditions. Variations in weather, planting environments, and subtle differences in the immediate surroundings of individual plants can lead to significant discrepancies in the time it takes for wheat to progress from the same growth stage to anthesis. Current image-based phenotyping approaches are limited in predicting individual wheat flowering times, highlighting the need for methods that integrate temporal dynamics and environmental variability.

This research innovatively leveraged relatively economical red, green, blue (RGB) image data combined with time-series weather data to develop a universal multimodal method for predicting the flowering time of individual plants. By employing RGB images, it analysed the development of each individual plant. This data was integrated with weather environmental factors, including temperature, rainfall, and sunlight duration, to address the specific requirements of predicting flowering time, particularly determining whether a plant will flower after a certain number of days. Since breeding and regulatory practices often require predicting critical days prior to actual anthesis, we have structured the flowering prediction challenge as a classification problem. To facilitate the model's application across diverse environments, particularly given the susceptibility of wheat flowering periods to environmental influences, we employ few-shot learning techniques that use metric-based learning, a type of similarity-based learning in computer vision [22]. This approach minimizes the need for extensive data collection. By engaging in meta-learning with large datasets and then utilizing smaller datasets to establish anchors, we enable the model to adapt to varying environmental conditions across different agricultural fields. The objectives of this study were threefold: First, to determine an appropriate data collection method, encompassing both plant images and weather data; second, to train a machine learning model that accounts for both plant images and weather data to predict flowering time at variable critical days to anthesis; and third, to generalise a few-shot learning model to be applicable to other fields with very limited samples.

## 2 Materials and methods

### 2.1 Experimental conditions for wheat plants

This research used 'Scepter', a Mid-season maturing wheat variety known for its significance in Australian commercial agriculture. Scepter is typically sown between late April and mid-May, and its time to anthesis varies with regional climatic conditions. Under standard field environments, flowering generally occurs between early and late September, requiring approximately four to five months from sowing to reach anthesis [23]. Due to the significant impact of environmental factors on flowering time, the experiment utilised a dual-environment approach, employing distinct datasets from each setting to develop separate models to predict flowering times, thereby validating the feasibility of the method. The first environment consisted of an outdoor semi-natural planting in large trays (Fig. S1a) using a 1:1:1 soil mixture (sand, clay, and UC mixture [24]), located at the Plant Accelerator, Waite Campus of the University of Adelaide (−34.971421°, 138.640039°). The trays (100 ​cm in length, 90 ​cm in width, and 45 ​cm in height) were systematically planted with wheat, with five rows per tray, six plants per row, with an inter-plant spacing of 8–10 ​cm. The second environment adopted a field trial, which was sown in a rain-fed clay-loam field located on the University of Adelaide's Waite Campus (−34.965530°, 138.634397°). Wheat was sown in two rows at 10 ​m in length, adhering to a row spacing of 15 ​cm and an inter-plant gap of 8–10 ​cm (Fig. S1b). This arrangement resulted in approximately 120 wheat plants, offering a field-based perspective on ‘Scepter's’ adaptive capacity and anthesis dynamics. During the tillering stage, urea fertilizer was applied at a rate of 30 ​kg/ha to support plant growth and development. To dissect the phenotypic and flowering time variability under different sowing seasons, the study was stratified by three strategically chosen sowing dates: Early (April 11, 2023), Mid (April 27, 2023), and Late (May 21, 2023). The initial two sowing periods were allocated to the outdoor semi-natural environment, each featuring two trays and culminating in four trays. This configuration yielded a comprehensive dataset of 60 plants to ensure replication for each of the Early- and Mid-sowing seasons; the Late sowing phase was conducted in the field. This diversified planting scheme, spanning outdoor semi-natural growth conditions and real field environments, and encapsulating a range of sowing times, was conceived to provide a holistic understanding of ‘Scepter's’ phenological behaviour and its flowering time adaptability in response to variable growth conditions.

### 2.2 Image acquisition

As wheat typically develops a main stem and multiple tillers, labelling and monitoring in this study occurred on the main stem. This approach allowed for precise documentation of the anthesis process for each plant, facilitating accurate anthesis predictions (Fig. S2). Image data collection was initiated as the labelled plants entered the flag leaf sheath opening stage (Zadoks growth scale Z47), a stage when the reproductive organs become visible as the boot swells and opens [25]. Image collection continued daily until the completion of the ear emergence stage (Z59). The visibility changes in the wheat's boot and head, which are key reproductive organs, were used as critical indicators for assessing wheat development and predicting flowering time. Following the image collection phase at each growth stage, the actual flowering time for each plant was recorded by allowing the plants to continue to grow to anthesis (Z60). RGB colour images were captured with an iPhone 13 Pro (iOS 16.5.1 version) in two formats: standard still images and 30-s videos, at a rate of 30 frames per second, both in 4K resolution. The still images had a resolution of 4032 ​× ​3024, while the videos were recorded at a resolution of 3840 ​× ​2160. The images were captured at varying angles between 45° and 75°, with a distance ranging from 30 to 50 ​cm to ensure the plant heads occupied approximately 20 ​% of the image frame. Video recording was employed to enhance the flexibility of image acquisition and minimise the need for manual focusing, thereby increasing the model's applicability in future scenarios. Despite a slight loss in resolution, the video maintained 4K image quality and offered improved data compression capabilities. The video data was subsequently converted to still images for analysis, applying the Laplacian Variance method to select the clearest frame per second from each video [26]. This method evaluates image sharpness by measuring the variance of pixel intensity gradients, with higher variance indicating sharper and more focused frames. This resulted in a daily collection comprising 15 still images and 30 video-derived images for each plant, with data acquisition occurring at varying times between 9 a.m. and 4 p.m. to ensure that the images captured a range of lighting conditions. This innovative method significantly enhanced the flexibility of image acquisition and minimised the need for manual focusing, thereby ensuring optimal clarity and detail in the documentation of plant development.

In this study, the data was divided into three temporal subsets—Early, Mid, and Late datasets—corresponding to the sowing groups previously defined. This stratification enabled a more precise analysis of phenotypic and flowering time variation across different sowing seasons. Due to the variation in sowing times, the plants experienced significantly different macro-environments (weather conditions), which influenced their growth, including from Z47 to Z59 and the number of days to anthesis. Table S1 outlines the characteristics of each dataset, including the number of imaged plants, the number of images taken, and the span of days before anthesis. The Early dataset spans a broader timeframe, capturing images from 23 days to 7 days before anthesis and includes images of 52 plants. The Mid dataset spanned over a relatively similar amount of time of 18 days–2 days before anthesis and had images of 57 plants. The Late dataset spanned a relatively short amount of time of 14 days–5 days before anthesis and had image data for 103 plants.

### 2.3 Weather record

Since time to anthesis in wheat is closely related to weather conditions, environmental data for each trial was recorded. Daily weather conditions for each plant, complemented by a 6-day forecast following the date of image capture, were recorded using data provided by the Australian Government's Bureau of Meteorology from Adelaide Airport SA weather station (station code: 023034) (Lat −34.5642°, Lon 138.3150°) [27]. The dataset included maximum and minimum temperatures (Celsius), sunlight duration (hours), and rainfall (mm). Both the daily photo-degree days (PDD) and the cumulative photo-degree days (CPDD) were calculated. The ‘PDD’ calculates the daily heat accumulation by averaging the day's temperature range and multiplying by the duration of sunlight (Formula (1)), while the ‘CPDD’ (Formula (2)) sums the daily PDD over ‘*n*’ days to track cumulative heat exposure [28]. Due to the absence of specific sunlight duration in the Bureau's forecasts, estimations were made based on 20-year monthly average sunlight durations. These were categorized according to conditions as 'sunny', 'mostly sunny', 'mostly cloudy', 'cloudy', and 'shower', corresponding to 100 ​%, 75 ​%, 50 ​%, 25 ​%, and 0 ​% of the average monthly sunlight duration calculated using the past 20-year historical data, respectively. This systematic integration of climatic data was designed to deepen the understanding of environmental effects on the growth and anthesis of wheat.

(1) $$Photo-degree\;days=\frac{\left(\mathrm{Max}\;temperature-\mathrm{Min}\;temperature\right)}{2}\times \text{Sunlight}\;\text{duration}$$

(2) $${Cumulative\;photo-degree\;days}_{n}={Photo\;degree}_{1}+{Photo\;degree}_{2}+\cdots +{Photo\;degree}_{n}$$

### 2.4 Extra dataset collection

In addition to the primary experiment, this study utilised a wheat field site in Rosedale, South Australia (Lat −34.544232°, Lon 138.835640°) as a test set. This test set will include weather data from an additional weather station to further validate the model's robustness and adaptability. Environmental data was collected from a weather station (coordinates: −34.51, 138.68, station code: 023021), which is 15 ​km away from the farm [27]. The field trials focused on Scepter wheat, the predominant variety at this site, constituting 87.5 ​% of the genetic material, and was sown on 31 May 2023. The site was prepared by deep ripping, followed by the application of 9.5 ​t/ha of gypsum and organic matter, then cultivated for planting. Cereal starter fertilizer (NPK 15-15-15) was used at 25 ​kg/ha, and herbicides Rexade and Amicide Advance 700 were applied at 100 ​g/ha and 0.5 ​L/ha at the 4–5 leaf and stem elongation stages, respectively. Due to restricted access to the Rosedale farm, image collection was limited to weekdays and conducted over a one-week period from 19 to 25 September 2023. During this period, developmental progress varied among individual plants as a result of micro-environmental differences. Consequently, even when plants appeared to be at similar growth stages, they experienced different weather conditions during the pre-anthesis phase. To maintain clarity and avoid excessive scope, the analysis was focused on a developmentally dynamic window, approximately corresponding to Z40 to Z45. This head-developing stage is particularly challenging for prediction, as individual plants exhibit high visual similarity despite underlying differences in phenological status. The dataset comprised images of 96 wheat plants and the methodology employed the same image capture techniques and analysis as described above. This independent dataset was not used for training or validation and was exclusively reserved as an external test set to assess model generalizability. It contributed to two major validation tasks. First, in the anchor transfer experiments, models trained on the Early, Mid, Late, or Full datasets were evaluated on the Rosedale set using anchors derived from different sources. Second, in the three-class classification task, the Late and Full models, using both Rosedale and Late anchors, were tested on the Rosedale dataset. These evaluations enabled an independent assessment of model transferability under field conditions and across varied sowing environments.

### 2.5 Model establishment

Each dataset (Early, Mid and Late) was independently trained to develop unique models, which were then cross-tested to evaluate their effectiveness. The entire methodology was referred to as few-shot learning and processed using Python, segmented into three distinct phases: (1) ‘Data Preparation’, where selective crops of the areas of interest in the images were made, and image-weather data combinations were constructed; (2) ‘Meta-Learning’, which employed a pairwise Siamese network to compare whether the days to anthesis of one plant was shorter or equal/longer than another by leveraging comprehensive image-weather data combinations; and (3) ‘Application to Few-Shot Learning’, which focused on using a limited number of plant images from different datasets to predict if a plant was within ‘*n*’ days of anthesis. By employing few-shot learning strategies, this study reduced the necessity for extensive data collection, enhancing the model's practicality and adaptability. This approach tested the model's adaptability across diverse datasets, ensuring its robustness and versatility. Detailed explanations of these processes are provided in the subsequent section.

#### 2.5.1 Data preparation

The primary transformations in wheat plants from Z47 to Z59 predominantly occurred in the wheat head, serving as the critical organ for assessing flowering time. To ensure a precise depiction of the individual plants’ developmental stages, the focus was directed solely on this region, as shown in Fig. S3. We employed the object detection model based on YOLO version 8 [29] to accurately identify the coordinates of the wheat head. The YOLO version 8 model was fine-tuned from a pre-trained baseline using a dataset comprising 4800 field images of wheat collected in 2022 (same location as the 2023 field), achieving a mean Average Precision of 0.88. Additionally, the heads appearing on non-main stems were manually excised from the cropped images due to the lack of flowering time records. Furthermore, to ensure image quality, images smaller than 20 ​× ​50 pixels (width and height) were removed. As a result, the Early-sown dataset comprised a total of 6783 photos of wheat heads on main stems, 8255 photos for Mid-sown, and 9998 photos for Late-sown.

**Fig. 1:**
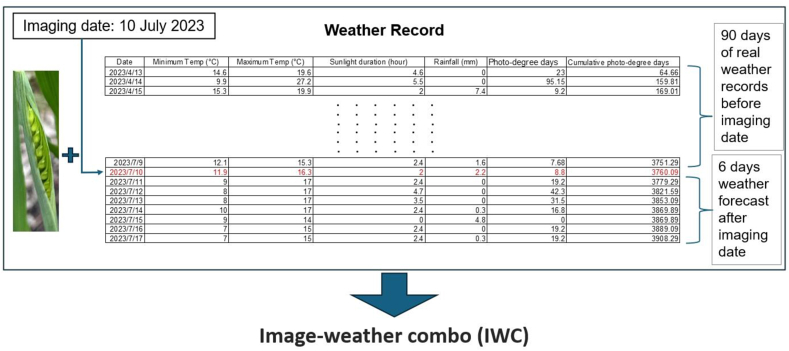
Image-weather data combo. A plant image merged with 90-day pre-imaging weather data and subsequent 6-day forecasts, enabling analysis of environmental impacts on growth. The red line indicates the imaging date of this picture.

The cropped wheat head images were combined with corresponding meteorological data to create the ‘Image-Weather Data Combo’ (IWC). This comprehensive dataset included detailed climatic information including minimum and maximum temperatures, sunlight duration, rainfall, and derived indices ‘PDD’ and ‘CPDD’ (Fig. 1). These elements captured the climatic conditions for the 90 days leading up to the imaging date and provided weather forecasts for the 6 days following, highlighting the critical influence of weather on plant flowering times. The choice of a 90-day period was based on the typical duration from sowing to reaching the flag leaf stage in spring wheat, which is about 90–100 days. The rationale for using a fusion of image and weather data stems from the recognition that the anthesis phase of a plant is influenced by both micro and macro-environmental factors. Image data provides insights into an individual plant's immediate conditions, capturing nuances like individual developmental stage. Meanwhile, weather data reflected broader environmental influences, such as temperature and precipitation amount. Such data combinations provide an expression of how individual plants grow under the combined effects of these environmental layers, facilitating accurate determination of flowering time. To optimise the model training process and minimise memory usage, both the image and weather data were converted into NumPy array formats. The data samples were then structured in a list format, with the first entry representing the image data and the second for the weather data.

#### 2.5.2 Meta learning framework

**Fig. 2:**
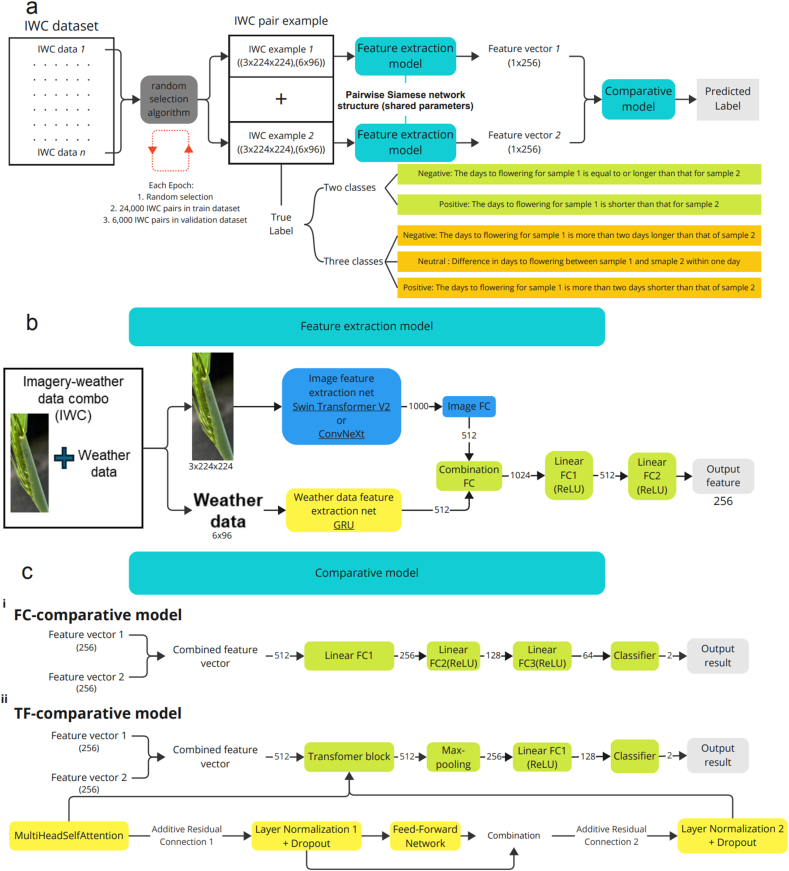
Training process in meta-learning framework. (a) illustrates model workflow through feature extraction and comparative analysis models for plant anthesis prediction in Meta-learning. (b) represents the feature extraction model which integrates Swin Transformer V2 or ConvNeXt network for extracting image features and a GRU network for mining weather features, culminating in a composite feature vector for subsequent tasks. (c) illustrates the architectural differences between two neural network models: the Fully Connected (FC) model (i) and the Transformer-based (TF) model (ii).

In this study, meta-learning served as a pivotal component, utilizing an extensive dataset that captured a comprehensive visual and climatic narrative of plant development. The objective was to train a model capable of precisely contrasting the developmental trajectories of two plant samples based on IWCs, which consisted of plant head images and synchronous weather records. This method enabled the model to effectively discern subtle differences in the wheat developmental stages influenced by both micro- and macro-environmental conditions, thereby enhancing its predictive accuracy. During the meta-learning training process, we established IWC pairs, with each pair containing two IWC samples. We randomly selected two IWCs from the dataset to form IWC pairs, assigning each pair a corresponding ‘negative’ or ‘positive’ label (Fig. 2 a). In this experiment, two classification methods were used: binary classification and three-class classification. For the binary classification, a ‘negative’ label indicates that the days to anthesis of the first IWC in the pair is equal to or greater than that of the second IWC, and vice versa. For the three-class classification, a ‘negative’ label indicates that the days to anthesis of the first IWC in the pair is more than two days longer than that of the second IWC; a ‘neutral’ label indicates that the difference in days to flowering between the first and second IWC is within one day; and a ‘positive’ label indicates that the days to anthesis for the first IWC is at least two days shorter than that of the second IWC. Theoretically, this arrangement allowed for up to *2*^*n*^ possible pairwise comparisons, with *n* representing the total number of IWCs in the dataset. This training approach significantly overcomes the limitations of limited datasets typically encountered in agriculture, not only enhancing the training process but also significantly improving the model's ability to generalise across different datasets, which is crucial for practical applications. A random data extraction algorithm was employed to randomly select two IWCs from the dataset to form sample pairs for model training (Fig. 2 a). Each training epoch utilised a dataset consisting of 24,000 training sample pairs, supplemented by 6000 validation sample pairs. This structured separation between the training and validation datasets facilitated effective model tuning and performance evaluation. A fixed seed in the random selection algorithm ensured consistency during the training and validation phases in every epoch. The random selection algorithm took into account the proximity of the images and flowering time. In 80 ​% of the pairings, the algorithm juxtaposed images with distinct flowering times, and 20 ​% of sample pairs involved images from the same days before anthesis. Moreover, to ensure a diverse representation of environmental and developmental conditions, the algorithm selected images from different IDs in 95 ​% of cases, significantly enriching the variability of the dataset and exposing the model to a broad spectrum of genetic and environmental interactions. Each independent dataset (Early-, Middle-, and Late-sown) underwent separate model construction and subsequent cross-testing. This included comparative tests using the independently trained models to assess the 6000 validation sample pairs from the other two datasets, as well as independent few-shot learning application tests. The Rosedale dataset (independent extra dataset) was reserved exclusively as an independent test set and not included in any training or validation steps. It was later used in the anchor transfer and three-class inference evaluations to assess the model's generalizability in novel environments.

After the IWC pairs were formed, our entire meta-learning process involved training a comparative model based on a Siamese network architecture and a binary classifier. The core characteristic of the 'feature extraction model' was the pairwise Siamese network architecture that included two instances sharing the same parameters (Fig. 2 a). The feature extraction model converted the individual IWC data in each IWC pair into one-dimensional (1D) feature vectors, which were abstract representations of the IWC in a lower dimension. Thus, an IWC pair would yield two 1D feature vectors after passing through the feature extraction model. These two vectors were then fed into the 'comparative model,' which played a critical role in determining the final prediction label. The comparative model analysed these feature vectors to determine the difference in days to anthesis between the two IWCs, categorising the relationship as shorter or equal/longer.

The IWC data first entered the feature extraction model, where the image information was processed by either Swin Transformer V2 or ConvNeXt (Fig. 2 b). The Swin Transformer V2 is renowned for its adaptive hierarchical designs and efficient attention mechanisms, making it ideal for tasks involving diverse scales [30]. On the other hand, ConvNeXt updates traditional convolutional designs by integrating principles from Transformers and CNNs, offering a balance of simplicity, scalability, and robust performance across various image-related tasks [31]. Both models, pre-trained on the ImageNet-1K V1 dataset [32] by the PyTorch [33] provided a solid foundation for transfer learning, utilizing 'base' version models with approximately 90 million parameters each. For processing the sequential weather data in IWC, Recurrent Neural Networks (RNNs) were used, specifically selecting the Gated Recurrent Unit (GRU) variant for its computational efficiency and ability to mitigate the vanishing gradient problem commonly encountered with traditional RNNs [34]. The GRU's architecture excels at capturing long-term dependencies in time-series data, making it an optimal choice for modelling complex weather patterns. After feature extraction, the image feature of 1000 dimensions was reduced to 512 dimensions through a fully connected layer (FC) and then combined with the weather feature of 512 dimensions to form a full feature vector of 1024 dimensions. Further, the data then passed through FC1 and FC2 to ultimately output a 256-dimensional feature vector. During this process, Rectified Linear Unit (ReLU) activations were interspersed [35] to introduce non-linearity (Fig. 2 b), facilitating the detection of intricate patterns. Notably, although pre-trained weights were used for feature extraction of the image information, the weights were not frozen during training; they were adjusted throughout the training process.

After extracting features from the IWCs, yielding 1D feature vectors, two vectors became inputs for the comparative model designed to evaluate disparities in flowering times between two samples. Two neural network architectures were developed as comparative models, one named 'FC-comparative model,' which was composed of fully connected layers, and another called 'TF-comparative model,' which was based on a transformer network structure (Fig. 2 c). It incorporated complex attention mechanisms and layer-wise transformations to discern the nuanced differences in the input features. The models took a 512-dimensional feature vector as input, which was composed of two feature vectors extracted from two IWC data through the feature extraction model, and then outputted a binary value to determine if one flowered earlier than another. In the FC-comparative model (Fig. 2 c (i)), the integrated input was processed by a series of four fully connected layers to gradually reduce the features. This architecture progressively refined the feature vectors, ultimately delivering them to a classifier layer for a binary classification task. ReLU activations [35] were introduced between the fully connected layers to introduce non-linearity for detecting complex patterns (Fig. 2 c (i)). In contrast, the TF-comparative model employed both a simplified and modified version of the Transformer architecture. As shown in Fig. 2 c (ii), it completely eliminated both the embedding layer and positional encoding and the core of the model also became relatively simple [36,37]. Specifically, it was composed of a single unit such as the Transformer block. The activation of the functions in the Transformer block can be divided into the following integrated steps. Transformer block processed data using a standard approach: initial multi-head self-attention with four heads discerns relationships, followed by a residual connection to counteract gradient issues. After that, a feed-forward network introduced non-linear parameters, while the second residual link contributed to stability [38]. The transformer output vector was then processed through a max-pooling operation, which selected the most essential features and reduced the dimensionality to 1-D, producing a highly concentrated and powerful vector. Finally, a fully connected dense layer was employed to reduce the overall number of features, culminating in a classification outcome.

The training process automatically incorporated early stopping to prevent overfitting. To enhance model convergence and generalizability, four core techniques were applied: cosine annealing for learning rate scheduling, exponential moving average (EMA) for stabilizing parameter updates, label smoothing to mitigate overconfidence in predictions, and a custom Adam optimizer adapted for this task. Cosine annealing helped the model escape local minima and improved overall convergence [39,40]. EMA smoothed parameter updates by weighting recent values more heavily [35,41]. Label smoothing reduced the risk of overfitting by softening categorical decision boundaries [39]. The Adam optimizer was further modified to improve training stability and better handle sparse gradients [42,43]. All implementation details, including hardware specifications and hyperparameter values, are provided in Table S2.

#### 2.5.3 Application of few-shot learning inference

**Fig. 3:**
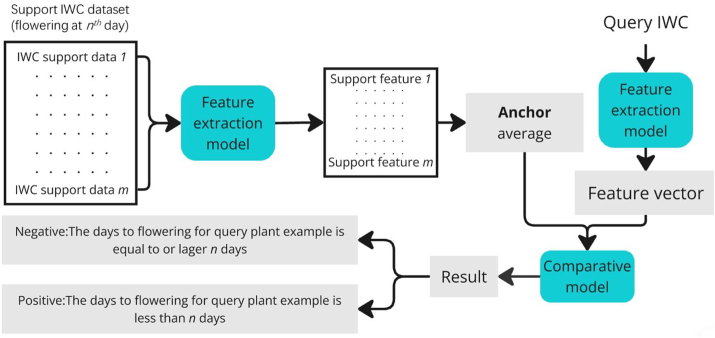
Few-Shot inference workflow. A schematic workflow illustrating the few-shot inference process for plant flowering prediction using the image-weather data combination. The feature extraction and comparative models were pre-trained through meta-learning and reused during inference without additional training.

For accurate prediction of the individual wheat flowering time in fields across different growth conditions where usually only a limited number of samples are available, we utilised principles derived from few-shot learning which needs only a small dataset that is directed to specific predictive tasks. To test the model in a testing field, the main approach involved establishing a standard anchor which was the averaged feature vectors of relatively small amounts of data representing the plants in the testing field that will flower after *n* days. Then the anchor is used to determine the flowering time of query samples, a particular individual wheat plant, by comparing whether the query sample will flower before or after this standard anchor (Fig. 3).To establish an anchor, a small support dataset of plants, which will flower after *n* days, needs to be collected, usually from previous years. The feature extraction and comparative models used here were pre-trained during the meta-learning phase and were reused in this inference step without additional training. Each IWC in this support dataset would be passed on to feature extraction model, which extracted meaningful features. These features were then averaged to form an anchor, which was a general profile of the feature that characterised plants near the anthesis stage. The query IWC will be processed through the same feature extraction to generate query feature vectors. The comparative model then evaluated whether the query plant will flower within *n* days by comparing the query vector to the anchor. A positive result means that the flowering time of the query sample will be within ‘*n*’ days, whereas a negative result implies it would need more than *n* days.

In this few-shot learning experiment, distinct critical days to anthesis were established to assess the adaptability of models to various tasks, taking into account specific model requirements and regulatory guidelines. According to Australian biosafety regulations, the critical day for flowering prediction is defined as 14 days to anthesis, while for pollination, it is set at 10 days. Bheemanahalli et al. [44] note that the peak timing for anthesis in wheat typically occurs early in the morning or late in the evening. However, our recordings were made exclusively at noon, potentially introducing a one-day deviation in the anthesis records due to this timing discrepancy. To address these challenges and ensure comprehensive model testing, five specific critical days to anthesis, 8, 10, 12, 14, and 16 days, were established. Both one-shot and few-shot methods were evaluated; specifically, for the few-shot scenario, we chose five plant images for each support dataset. This selection was informed by our observation that the five-shot results significantly outperformed the three-shot approach and were comparable to the ten-shot method. The five-shot anchor was constructed from 30 to 50 photos of five plants. Additionally, we implemented ten-cycle tests, i.e., testing one-shot or five-shot learning ten times in each critical day by randomly selecting ten different datasets from the data pool to establish anchors and queries. The performances for each critical day were then averaged, offering a detailed evaluation of each specific requirement and mitigating the impact of outliers. The construction of anchors and queries were set so that the ten-cycle selection of plant images in the same day was random, while the selected plants were kept the same across different days. This approach reduced the variability due to the construction of anchors and queries from different plants across different days and focused on the comparison the capabilities of different models.

#### 2.5.4 Evaluation method

To evaluate model performance, we adopted the F1-score as the primary evaluation metric. Given that this study focuses on identifying whether individual wheat plants will flower within a defined set of critical days to anthesis, rather than predicting the exact date, we reformulated the task as a binary classification problem: determining whether flowering will occur within a predetermined time window. This formulation aligns with the practical needs of breeding programs, where timely flowering prediction within key windows is essential for synchronizing pollination, planning field operations, and ensuring regulatory compliance. Accordingly, the F1-score is particularly appropriate for assessing this type of binary classification outcome, as it considers both precision, the ability of the model to avoid false positives, and recall, the ability to detect true flowering events. It is defined as the harmonic mean of precision and recall in Formula (3):

(3) $$F1-score=2\frac{TP}{2TP+FP+FN}$$

where *TP* is the number of true positives (correctly predicted flowering within the critical window), *FP* is the number of false positives (flowering incorrectly predicted within the window), and *FN* is the number of false negatives (missed flowering within the window). Unlike continuous regression metrics such as RMSE, the F1-score more accurately reflects model performance in distinguishing biologically meaningful flowering windows.

## 3 Results

### 3.1 A statistical analysis of anthesis

**Fig. 4:**
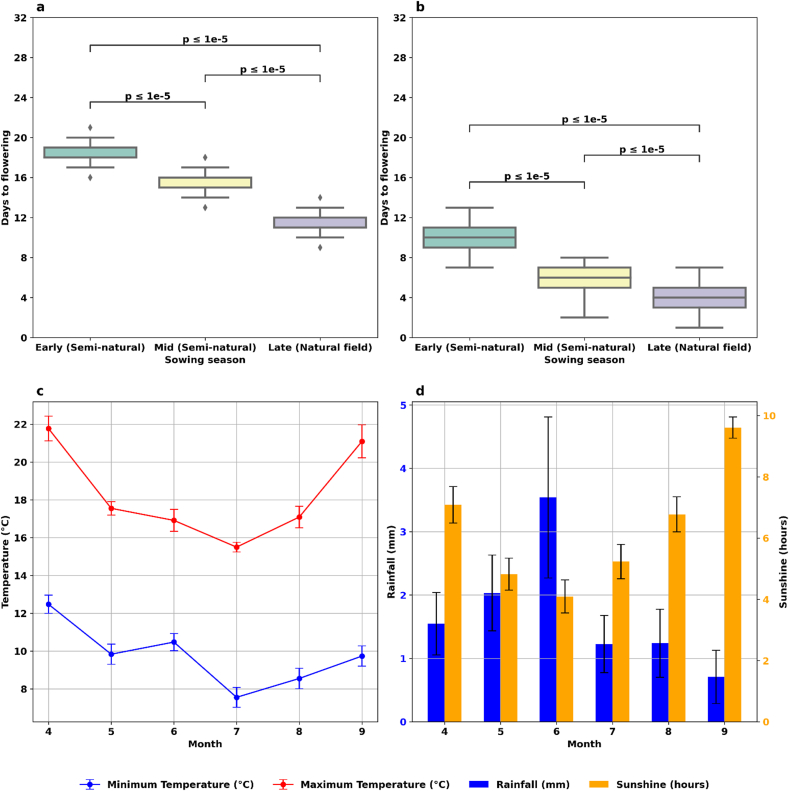
Days to anthesis across different sowing seasons, with monthly weather data for the 2023 growing season. Panel (a) & (b) illustrate the days to anthesis from two growth stages across three sowing seasons: Early (sown on 11 April 2023), Mid (sown on 27 April 27, 2023), and Late (sown on 21 May 2023). (a) shows the days from Z47 to anthesis; (b) shows the days from Z59 to anthesis. (c) displays a bar and line graph of the monthly changes in average maximum (red line) and minimum daily temperatures (blue line) in Celsius. (d) presents average daily sunlight duration in hours (orange bars) and average daily rainfall in millimetres (blue bars) from April to September, sourced from the Adelaide Airport SA weather station. Weather results are presented as the mean ​± ​standard error of the mean (s.e.m.).

As was expected, different growing macro-environmental conditions affected the flowering time of wheat (Fig. 4 a, b). Significant differences in the flowering time of the plants across multiple sowing conditions (*P* ​≤ ​0.001, ANOVA with Tukey's Honestly Significant Difference Test, details in Table S3). Wheat sown Early in outdoor semi-natural conditions required an average of 18.4 days (±0.153 ​s.e.m.) from Z47 to anthesis, whereas those sown in Mid-season conditions took an average of 15.7 days (±0.145 ​s.e.m.) to anthesis (Fig. 4 a). There was a marked distinction between wheat sown in outdoor semi-natural conditions (Early- and Mid-seasons) and those sown in Late-season field conditions, the latter taking 11.6 days (±0. 0.107 ​s.e.m.) from Z47 to anthesis. Average days from Z59 to anthesis was 10.0 ​± ​0.191 ​s.e.m., 6.1 ​± ​0.202 ​s.e.m., and 4.1 ​± ​0.140 ​s.e.m. days for Early sown, Mid sown and Late sown wheat, respectively (Fig. 4 b). The 2023 growing season was representative of a dry season in South Australia with lowest temperatures experienced in July, and minimum day length in June (Fig. 4 c). Both temperature and daylength increased from July to the end of the experiment. Rainfall peaked in June 2023 with an average of 3.54 ​mm per day (Fig. 4 d). The increase in maximum temperature and sunlight result in shorter flowering times in the Late sowing season. This complex interplay between growth stages, weather conditions, and sowing times posed a challenge to the simplistic use of development stages alone to predict anthesis.

### 3.2 Binary classification cross-validation result

**Fig. 5:**
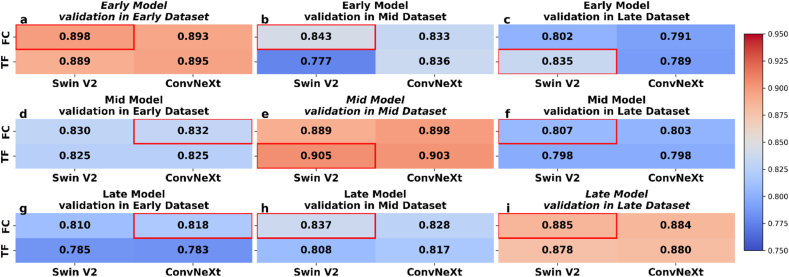
Cross-validation of the Early, Mid, and Late models using F1 score. Each panel displays validation results for the models trained on different datasets Early (a, b, c), Mid (d, e, f), Late (g, h, i) and validated across various other datasets. The red boxes highlight the highest F1 scores each model and validation set. *Italic text* indicates that the training and validation data came from the same dataset.

The datasets vary in terms of size and conditions of data collection (Table S1), which could potentially affect model performance when tested across different datasets. To evaluate the generalizability of the meta-learned models prior to applying them in the few-shot learning stage, we first conducted a cross-dataset validation. In this process, each model was trained and validated on a single dataset (Early, Mid, or Late), and subsequently tested on the remaining two datasets using IWC pairs. This strategy enabled the three datasets to function as mutually independent test sets, allowing for a systematic evaluation of the models’ ability to compare IWC pairs under diverse seasonal and environmental conditions. The heatmaps display the validation performance of each different model (Early, Mid, Late) in different datasets across multiple seasons and field conditions. It shows that the models had overall good performance by using the incorporation of the Swin V2 and ConvNeXt architectures with either FC or TF comparative designs. When the models were validated on the same dataset on which they were trained, the F1 scores were all above 0.85, some of them even over 0.90 (Fig. 5a–e and i). When the models were cross-validated on different datasets, the F1 scores typically exceeded 0.75, with most approaching or surpassing 0.80. This high level of accuracy across various conditions highlights the effectiveness of the models. These results demonstrated a relatively complicated relationship between the type of model architecture, dataset specifics and the results of their validation. Within each dataset, performance differences across model architectures were relatively small, with F1 scores typically exceeding 0.80 across most configurations (Fig. 5). For instance, on the Early dataset, Swin V2 and ConvNeXt achieved F1 scores of 0.830 and 0.832, respectively, both slightly outperforming the TF baseline of 0.825 (Fig. 5d). On the Late dataset, Swin V2 with FC reached 0.807 (Fig. 5f), while ConvNeXt with FC reached 0.818 on the Early dataset (Fig. 5g). These results suggest that FC designs marginally outperformed their TF counterparts, however, the overall differences between feature extractors and comparative modules were minor. In contrast, the sowing group itself had a more pronounced impact. Models generally performed better when tested on datasets with sowing times closer to their training set. For instance, the Early model achieved an F1 score of 0.843 on the Mid dataset, compared to 0.835 on the Late dataset (Fig. 5b and c). Similarly, the Late model reached 0.837 on the Mid dataset, outperforming its 0.818 result on the Early dataset (Fig. 5h–g). These findings reinforce that during the meta-learning validation phase, model architecture had relatively limited influence on predictive performance, whereas differences in sowing time and environment, captured by the dataset, were the dominant factors affecting cross-dataset outcomes.

### 3.3 Few-shot learning inference

#### 3.3.1 Binary classification one-shot inference result

**Fig. 6:**
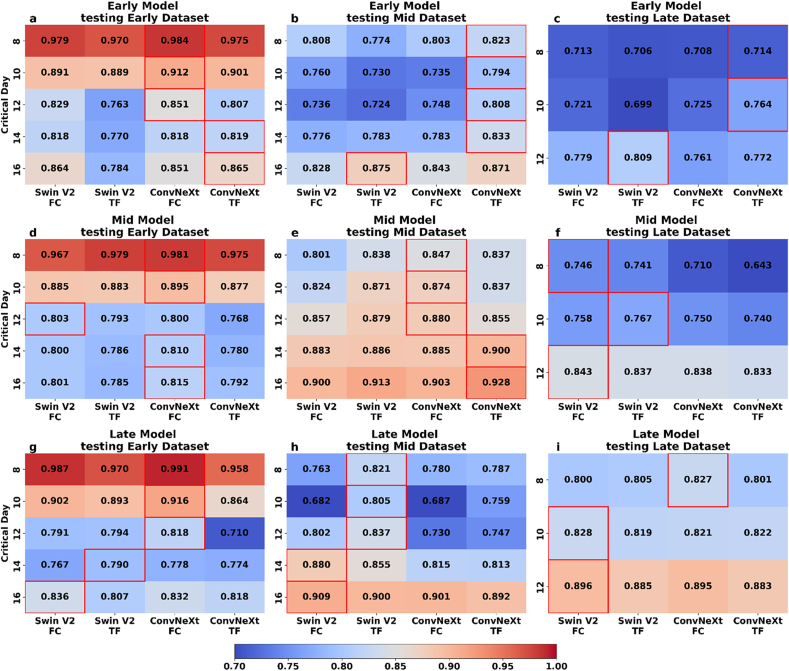
Heatmap of model performance (F1 score) on one-shot learning application in flowering prediction. This heatmap displays performance metrics for four neural network structures, Swin V2 and ConvNeXt, both with FC and TF variations, across three datasets corresponding to Early, Mid, and Late datasets. Subfigures ‘a, b, c’ represent the Early model, ‘d, e, f’ represent the Mid model, and ‘g, h, I’ represent the Late model. The red boxes highlighted the highest F1 scores in the group.

As shown in Fig. 4, the distributions of days to flowering vary not only between the Early, Mid, and Late datasets, but also within each individual dataset. This intra- and inter-dataset variability reflects real-world heterogeneity and provides a meaningful basis for evaluating the robustness of one-shot learning models across different critical days to flowering under diverse testing conditions. Fig. 6 presents the F1 scores from one-shot learning inference experiments using Early, Mid, and Late models, each trained on its respective dataset and tested on the remaining two datasets. This mutual cross-testing strategy, conducted during the inference stage rather than during training, enabled a robust evaluation of model generalization under different sowing conditions. In the Early dataset's results, where the training and testing datasets were identical, ConvNeXt models showed strong performance when evaluated on the same dataset used for training (Early dataset). ConvNeXt-based model achieved F1 scores above 0.85 across all critical days, peaking at 0.984 on day 8 (Fig. 6 a). In terms of the Mid dataset (Fig. 6 b), Swin V2 (TF) achieved the highest F1 score only on day 16 to anthesis (0.75), after which ConvNeXt (TF) consistently outperformed it across all remaining critical days to anthesis. ConvNeXt (TF) reached a peak F1 score of 0.833 on day 14, maintained strong performance on day 12 (0.808), and remained above 0.79 on both day 10 and day 8. In the Late dataset, on 12 days to anthesis, Swin V2 (TF) achieved the highest F1 score at 0.809. ConvNeXt (TF) led on the 10 days with an F1 score of 0.764 and 0.714 on the 8 days to anthesis (Fig. 6 c). Therefore, ConvNeXt models demonstrated a relative advantage, outperforming Swin V2 in most scenarios across different critical days to anthesis. Overall, the performance of ConvNeXt models, particularly the TF design, across all testing situations and durations was consistently strong. These conclusions emphasize the robustness of the ConvNeXt (TF) structure in managing diverse and challenging environments, clearly observable in the testing of Early models within the Mid and Late datasets.

In the Early dataset testing of the Mid model, results for days further from anthesis, F1 scores were lower on earlier days (16 and 14 days to anthesis), averaging around 0.8, with Swin V2 models generally outperforming ConvNeXt (Fig. 6 d). On day 12, both models performed similarly, with Swin V2 at 0.803 and ConvNeXt (FC) at 0.800. From day 10 onward, all models exceeded 0.85, with ConvNeXt (FC) leading, achieving 0.895 on day 10 and peaking at 0.981 on day 8. (Fig. 6 d). In the Mid dataset, where the model was tested under identical conditions to its training, ConvNeXt consistently showed superior performance across all critical days to anthesis (Fig. 6 e). ConvNeXt (TF) achieved the highest F1 scores in the Mid dataset, leading with 0.928 on the 16 days, and 0.900 on the 14 days. On days 12, 10, and 8, the FC design outperformed TF, recording F1 scores of 0.880, 0.874, and 0.847, respectively. In the Late dataset, despite significant deviations from the training setup, the models had adaptability, with Swin V2 models often leading in scores (Fig. 6 f). On the 12 days to anthesis, FC regained the lead, showcasing its strength with a score of 0.843. By the 10 days, TF had the highest score of 0.767. On the 8 days, TF and FC scored similarly, with 0.741 and 0.746 respectively. Overall, the ConvNeXt (FC) showed strong performance in the Early and Mid datasets, particularly under matched training and testing conditions. However, its advantage diminished in the Late dataset, where Swin V2 demonstrated greater adaptability to divergent conditions. This highlights the varying robustness of ConvNeXt (FC) across testing scenarios, especially under challenging anthesis prediction windows.

In the Early dataset for the Late model, all configurations had robust performance, with F1 scores generally surpassing 0.9 in the days closest to anthesis (Fig. 6 g). ConvNeXt (FC) achieved the highest scores on day 8 (0.991) and day 10 (0.916). Performance declined in earlier days, with ConvNeXt (FC) leading on day 12 (0.818), and Swin V2 (TF) on day 14 (0.790). By day 16, scores slightly increased, with Swin V2 (FC) and ConvNeXt (FC) both scoring above 0.83. In the Mid dataset, Swin V2 TF consistently outperformed the ConvNeXt models from the 8 to the 12 days to anthesis, with a very clear advantage, typically exceeding other models by 0.05–0.1. It led with an F1 score of 0.821 on the 8 days (Fig. 6 h). This trend was maintained through the 10 and 12 days with scores of 0.805 and 0.837, respectively. By the 14 and 16 days to anthesis, Swin V2 (FC) became the dominant model configuration, recording scores of 0.909 and 0.900 respectively. The result of the Late dataset (Fig. 6 i), where the testing and training conditions were identical, ConvNeXt (FC) recorded the highest initial F1 score on the 8 days to anthesis at 0.827. By the 10 days, Swin V2 (FC) established a clear advantage with an F1 score of 0.828. On 12 days, while all model results were close, around 0.89, Swin V2 (TF) achieved the highest score at 0.896. Throughout the testing phases for the Late model, ConvNeXt (FC) initially dominated with the highest F1 scores in Early stages but was eventually overtaken by Swin V2 models, which showcased stronger performance in Later stages and under consistent training and testing conditions. Swin V2 (TF) particularly excelled in the Late dataset, achieving the highest F1 score by the 12 days, highlighting its adaptability and effectiveness in varied testing scenarios.

Across all one-shot learning scenarios, ConvNeXt (FC) exhibited strong performance under matched training and testing conditions, particularly in the Early and Mid datasets. However, Swin V2, especially the TF design, demonstrated greater adaptability in the Late dataset and under cross-dataset testing, achieving leading F1 scores on several challenging prediction days. These results highlight the trade-off between stability and adaptability in model performance across diverse phenological and environmental conditions.

#### 3.3.2 Binary classification five-shot inference result

**Fig. 7:**
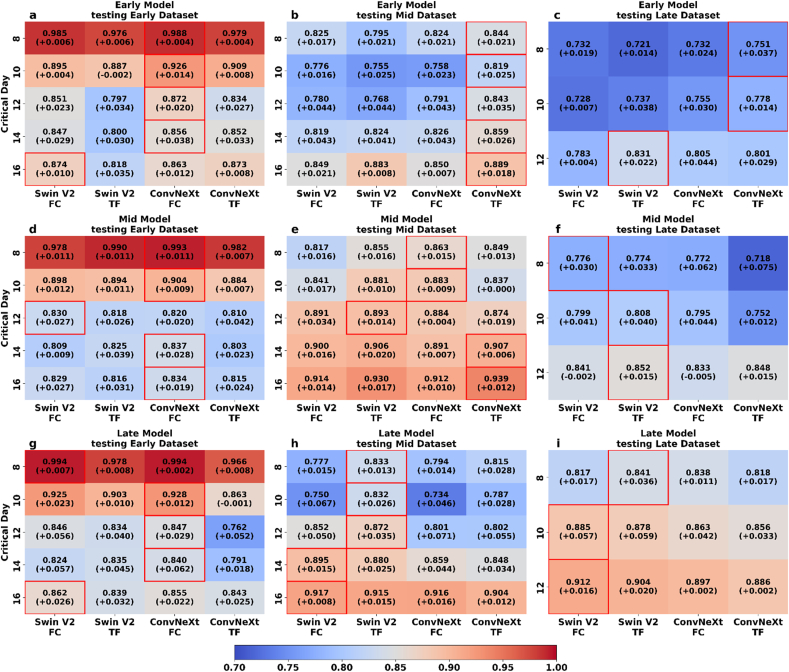
Heatmap of model performance (F1 score) from one-shot to five-shot learning in flowering prediction. Each cell displays the F1 score achieved by the five-shot learning, with the value in parentheses indicating the improvement relative to the corresponding one-shot learning. The heatmap compares four neural network architectures, Swin V2 and ConvNeXt, both with FC and TF variations, across three datasets corresponding to Early, Mid, and Late datasets. Subfigures 'a, b, c' represent the Early model, 'd, e, f' represent the Mid model, and 'g, h, i' represent the Late model.

Compared to one-shot learning, five-shot learning resulted in consistently improved model performance across all datasets, with most F1 scores exceeding 0.80, the satisfactory level in Fig. 7 [45]. For the Early model (Fig. 7 (a, b, c)), the dominant model did not show significant changes from the 8 to the 14 days to anthesis. For example, the ConvNeXt (FC) remained the dominant model with an F1 score exceeding 0.850 in Early dataset testing result. The only change was on 16 days to anthesis, where Swin V2 (FC) led with the highest value, achieving an F1 score of 0.874. In the Mid dataset, model rankings remained consistent from days 14 to 8, with ConvNeXt (TF) continuing to dominate. However, F1 scores improved by around 0.03 across these days. Notably, the top model on day 16 shifted from Swin V2 (TF) in one-shot to ConvNeXt (TF) in five-shot, with a substantial increase in F1 from 0.75 to 0.889. For the Late dataset, the increase in F1 scores was evident; on the 8 days to anthesis, the highest F1 score in one-shot learning was 0.714 by ConvNeXt (TF), and in five-shot learning, the highest score in the same model was 0.751, reaching an acceptable level [45]. Moreover, on 10 and 12 days, scores rose above 0.75. In terms of the five-shot learning results for the Mid model, depicted in Fig. 7 (d, e, f), the Early dataset showed a consistent trend where ConvNeXt (FC) remained the dominant model, maintaining F1 scores above 0.80 except on 12 days to anthesis where Swin V2 (FC) leads with an F1 score of 0.830. For the Mid dataset, there was a modest improvement in F1 scores. The leading model configuration remained largely unchanged, the only change occurred on 12 days to anthesis, where the dominant model shifted from ConvNeXt (FC) in one-shot learning results to Swin V2 (TF) (0.893). In the Late dataset, Swin V2 remained the dominant model, with most models exceeding an F1 score of 0.75 on 8 days, and Swin V2 (FC) continued to be the top model. Notably, on 12 and 10 days, the dominant model moved to Swin V2 (TF). For the Late model, the dominant model showed no significant change in the Early dataset, except for the 14 days to anthesis where the top model shifted from one-shot's Swin V2 (TF) to five-shot's ConvNeXt (FC) (Fig. 7 g, h, i). However, the results for both models in five-shot learning were close, with F1 scores of 0.835 for Swin V2 (TF) and 0.840 for ConvNeXt (FC). Additionally, from 16 to 10 days to anthesis, only ConvNeXt (TF) was below 0.8. The Mid dataset displayed modest improvements with no significant increases in performance. The most notable change was that even non-dominant models generally achieved F1 scores above 0.75. In the Late dataset, the dominant model changed only on 8 days, where the top model shifted to Swin V2 (TF) with an F1 score of 0.841.

In this experiment, we selected several different critical days to anthesis for few-shot learning tests. As the plant growth stages change, the F1 score itself also varied. The scores in the Early dataset mostly remained above 0.80 in both learning scenarios, with a slight decline from 16 to 14 days to anthesis, followed by a significant upward trend from 14 to 8 days, with the most pronounced increase from 12 to 8 days. The Mid dataset initially showed a slight decline in F1 scores, but a slight upward trend began as it approached 10 days to anthesis. The Late dataset, however, exhibited a consistent downward trend in F1 scores, with the closer to anthesis, the lower the F1 scores, suggesting that the model struggled more with the late growth stage data, leading to lower accuracy as anthesis approached. In general, the Early model showed improvement as it approached the flowering stage, especially evident in the Early dataset, while the Mid model's performance improved closer to flowering but started lower initially. The Late model displayed decreasing performance as the flowering stage neared. This analysis revealed that the model's performance was influenced by the growth stage of the plant data, with Early dataset yielding higher and more stable F1 scores, Mid dataset showing improvement closer to anthesis, and Late dataset posing challenges, resulting in declining model performance.

#### 3.3.3 Ablation study on weather integration

**Fig. 8:**
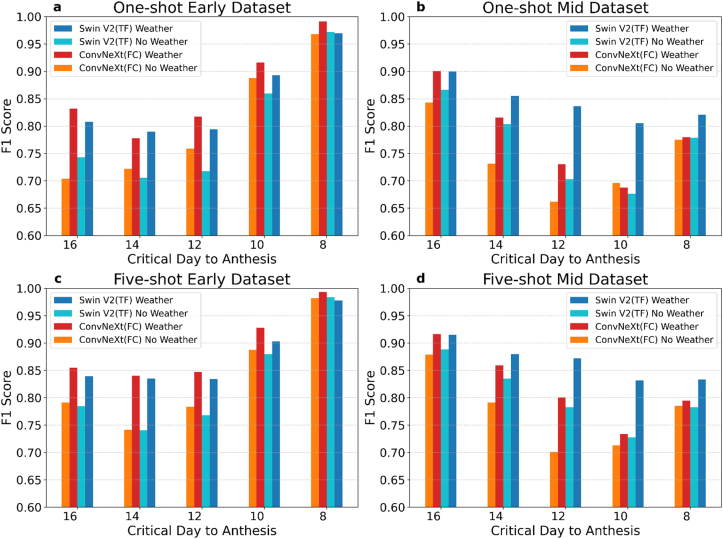
Performance comparison of models under weather and no-weather conditions. The charts compare the F1 scores of Swin V2 (TF) and ConvNeXt (FC) models trained from Late dataset and then tested in Early (a, c) and Mid-datasets (b, d) across one-shot and five-short learning, highlighting the impact of weather conditions on model performance.

One of the important innovations of this experiment was the multimodal model design, which allowed a single model to analyse both weather and images simultaneously and combine them to produce an output. To understand the impact of weather itself on model output, we selected the Late dataset and used the Swin V2 with TF neural network combination to train a model without weather input to test the impact of weather on model output (Fig. 8). Additionally, we chose the ConvNeXt (FC) combination to enhance the reliability of the experimental results. In the one-shot learning results, apart from the 8 days to anthesis in the Early- and Mid-datasets, the performance gap between the models under weather and no-weather conditions widened significantly from 16 to 10 days to anthesis (Fig. 8 a, b). For example, in the Early dataset testing, ConvNeXt (FC) achieved a performance of 0.78 with weather data, compared to 0.72 without weather data on 14 days to anthesis (Fig. 8 a). This improvement highlights the positive impact of including weather data, as it led to a more reliable model performance. The largest gap was observed with Swin V2 (TF), which showed a performance of 0.79 with weather data versus 0.71 without weather data on 14 days in the Early dataset testing. In the Mid dataset testing, the largest difference occurred on 12 days to anthesis, where Swin V2 (TF) improved from 0.71 in no-weather conditions to 0.84 with weather data, and ConvNeXt (FC) improved from 0.69 to 0.80. Additionally, the inclusion of weather data had a more significant impact on ConvNeXt (FC) in the Early dataset, while Swin V2 (TF) saw greater improvement in the Mid dataset (Fig. 8 a, b). Notably, on 8 days to anthesis in the Mid dataset, Swin V2 (TF) was the only model to exceed 0.8. For the five-shot learning results, the conclusions were similar to those from the one-shot results (Fig. 8 c, d). From 16 to 10 days to anthesis, the inclusion of weather data significantly improved model accuracy. The key difference is that the five-shot results were consistently higher than the one-shot results. Furthermore, majority of models with the inclusion of weather factors in our experiment performances exceed 0.8 over satisfied level, showcasing the beneficial impact of weather conditions.

#### 3.3.4 Anchor transfer from binary to three-class tasks

**Fig. 9:**
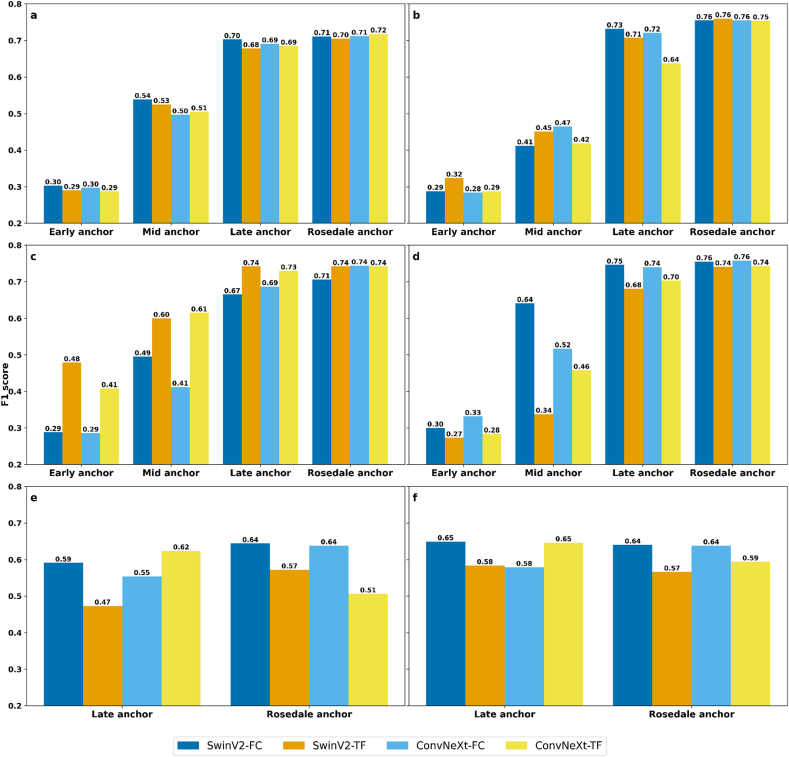
Comparison of F1 scores from few-shot learning inference for flowering prediction tasks on the Rosedale test dataset. Panels (a, b, c, d) show results from two-class classification models trained using the Early (a), Mid (b), Late (c), and Full (d) datasets, with each model evaluated using anchors derived from Early, Mid, Late, and Rosedale data. Panels (e) and (f) display results from three-class classification models trained on the Late and Full datasets, respectively, evaluated using anchors from Late and Rosedale data.

To simulate a realistic deployment scenario in plant breeding, this study evaluated the ability to apply pre-trained meta-learning models and historically constructed anchors to predict flowering in new environments. The framework assumes that, if environmental conditions, particularly sowing time and weather dynamics, are sufficiently similar, existing anchors from past trials can be reused without collecting additional local samples or retraining the model. This would eliminate the need for both large-scale model retraining and even small-sample anchor reconstruction, significantly reducing the burden of field deployment. In this study, four different anchors, derived from the Early, Mid, Late, and Rosedale datasets, were used with each of the Early, Mid, Late, and Full (combination Early, Mid, Late) models to predict the Rosedale dataset. As shown in Fig. 9 (a, b, c, d), anchors generated from the Late dataset produced nearly identical F1 scores to those of Rosedale-specific anchors, particularly in the Mid, Late, and Full models. In contrast, anchors derived from the Early and Mid datasets resulted in substantially lower performance. This is likely because the sowing date of the Rosedale dataset occurred only 10 days later than that of the Late dataset, and the field site is geographically close to Adelaide, resulting in similar climatic conditions. Such environmental similarity supports the strong transferability of the Late anchor to the Rosedale setting. In terms of model architecture, ConvNeXt (FC) consistently achieved the highest and most stable F1 scores across settings. In the Full model, ConvNeXt (FC) reached 0.76 with the Rosedale anchor and 0.74 with the Late anchor, underscoring its robustness. In addition to binary classification, we extended our framework to a more fine-grained three-class flowering prediction task, where the model distinguishes whether anthesis occurs (1) before, (2) after, or (3) within one day of a predefined critical date. Building on the consistent performance of the Late and Full models in the two-class evaluation, and their demonstrated suitability for meaningful comparison with the independent Rosedale test set, we selected these two models for the three-class scenario. Additionally, only the Late and Rosedale anchors were used, as the Late anchor had already shown strong transferability, likely due to its sowing date being just 10 days apart from Rosedale and its geographical proximity to Adelaide, which led to similar climatic conditions. The introduction of a third class increased the complexity of the task and resulted in an overall reduction in model accuracy compared to the binary setting. No F1 scores exceeded 0.75, yet most configurations still achieved values above 0.6, indicating a viable level of predictive accuracy under more stringent classification constraints. As shown in Fig. 9 (e, f), ConvNeXt (FC) consistently outperformed other configurations. In the Late model, ConvNeXt (FC) achieved an F1 score of 0.62 with the Late anchor and 0.64 with the Rosedale anchor. Swin V2 (FC) showed comparable results with 0.59 and 0.64, respectively. In the Full model, ConvNeXt (FC) again achieved the top performance, 0.65 using the Late anchor and 0.64 using the Rosedale anchor, highlighting its robustness and generalizability. Based on findings from both the two-class and three-class scenarios, increasing the training dataset size had limited impact on improving model accuracy. In contrast, using a reliable anchor derived from an environmentally aligned dataset played a much more significant role in determining predictive performance.

## 4 Discussion

### 4.1 Weather integration analysis

The integration of plant imagery and weather data was found to improve model accuracy (Fig. 8), highlighting the value of incorporating environmental context into phenological prediction. Plant growth is influenced by continuous soil and environmental factors and exhibits certain time-series characteristics, for example longer sunlight duration and higher temperature can advance the flowering time of wheat [46]. Therefore, integrating weather data allows models to capture temporal dynamics affecting crop development. Additionally, weather data typically has relatively coarse spatial resolution for whole field wheat growth conditions, and imagery provides comprehensive information on individual wheat. This enables models to refine the spatial generalization of weather data using high-resolution RGB imaging, thereby improving the accuracy and applicability of predictions [47]. Additionally, it is worth noting that results for those days closer to flowering, such as 8 and 10 days, showed minimal differences in the Early dataset, and even in the Mid dataset where the differences were not very pronounced. However, the largest improvements were found between 12 and 16 days, which directly correlated to the growth stage of the plants. During the period from 8 to 10 days to flowering, wheat plants have already developed a complete head and are at full-head stage. However, from 12 to 16 days, the wheat heads are still in fast developing stage, with much of it still covered by the leaf sheath. The wheat at head growth stage (Z47 to Z57), which lacks distinct graphic features, did benefit from the integration of weather data [46]. Therefore, this integration provided time-series environmental details that impacted crop growth enhancing the accuracy of flowering time prediction.

### 4.2 Image feature extraction model (Swin V2 and ConvNeXt) comparison

Using different image feature extraction networks, such as Swin V2 or ConvNeXt, resulted in varied performance across the three models trained on different datasets (Fig. 6, Fig. 7). The advantage of each image feature extraction model depends on the training dataset employed. To further investigate how these networks extract and process visual information, we applied Gradient-weighted Class Activation Mapping (Grad-CAM), a visualization technique that helps interpret the focus of image feature extraction networks by highlighting the key regions in an image that contribute to predicting a specific outcome. This enables gradients of any target output flowing back to the final convolutional layer to be captured, thereby producing a heatmap that emphasizes the significant areas in the model's decision-making process [48]. We referred CAM methods in the PyTorch library for Transformer-architectures models (Swin V2) since such models do not follow convolutional neural networks design and are required to reshape the final output layer to finish with visualization [49]. We focused on the Mid-dataset, according to the results in Fig. 6 (b and h) and 7 (b and h), Swin V2 (TF) shows a clear advantage in the Late model, while ConvNeXt (TF) performs better in the Early model. By visualising the test data from the mid dataset, we compared the impact of these two feature extraction models on the F1 score.

**Fig. 10:**
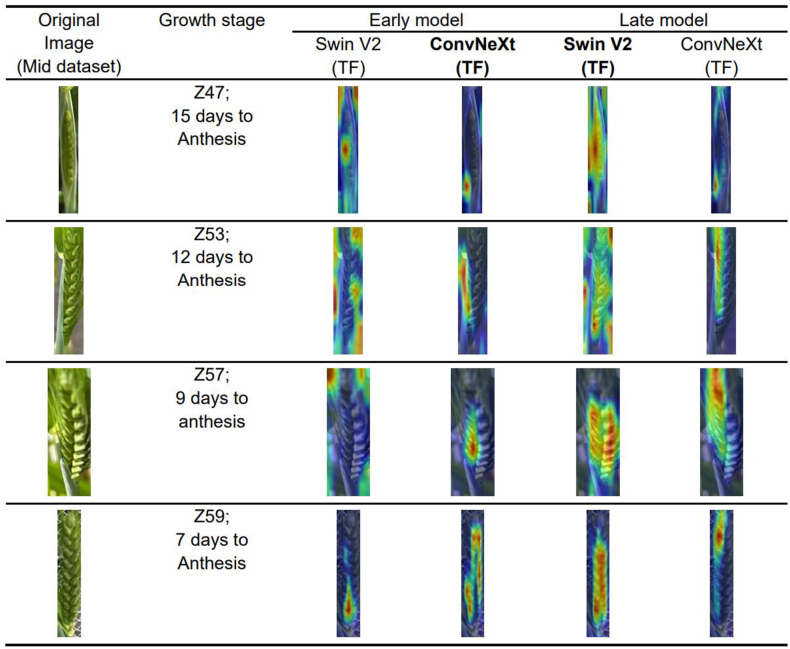
Grad-CAM heatmap visualization for feature extraction model interpretability. The heatmap compares the significant areas influencing the decision-making of Swin V2 (TF) and ConvNeXt (TF) between the trained early and late models tested in the mid dataset. The selected images were from different growth stages of wheat from Z47 to Z59. The bold text represents the advantageous image feature extraction neural network structure for each model. ConvNeXt is the preferred network for the Early model, while Swin V2 is preferred for the Late model.

In the early model comparisons between ConvNeXt and Swin V2, ConvNeXt consistently outperformed Swin V2, with this advantage being particularly pronounced from 8 days to 14 days to flowering. By 16 days to flowering, however, the performance difference between the two models diminished significantly. During the boot swollen stage (Z47) —approximately 16 days to flowering—neither model emphasized the plant regions significantly. During the head developing (Z53 to Z57) and full head stages (Z59), although Swin V2 displayed a broader activation area indicative of a generalized approach, it did not accurately focus on specific plant regions (Fig. 10). In contrast, ConvNeXt exhibited clearer, stronger activations, precisely targeting key features specifically located at the root sheaths surrounding the emerging wheat spikelets and the spikelets themselves. Visual outputs show that ConvNeXt adeptly captured these vital transitional growth phases. In contrast, the late model of Swin V2 was more accurately concentrated on key plant feature areas (Fig. 10). Notably, the most significant change was observed during the boot swollen stage (Z47), where Swin V2 targeted the wheat boot swelling area with high accuracy. Swin V2 in late model consistently displayed greater focus and clarity in heatmap activations across different stages of growth. Furthermore, as plants reached the full head stage (10 or 8 days to anthesis), the focus of the imagery became more pronounced, exerting a greater impact on the model's overall outcomes.

Notably, the performance of the late model from 16 to 12 days to flowering was demonstrated to be superior to that of the early model, as illustrated in Fig. 6, Fig. 7. Comparing the Early and Late models, both Swin V2 and ConvNeXt in the Late model focus on more areas than in the Early model, primarily because the Late model's training set contains more image data (Table S1), leading to a larger dataset. Considering the design characteristics of the models, the transformer-based Swin V2 requires a larger dataset of image data to perform optimally [50]. Moreover, Fig. 10 shows that as the full heading stage progresses, the image information became increasingly critical in guiding the model's data results. This is consistent with observations in Fig. 9, which show that as plants advance to the full head stage, their distinct morphological changes from earlier stages (16–12 days to anthesis) enhance the significance of image features in the entire model. In summary, the quality of the results is closely linked to the focal areas of the image feature extraction model, and the greater the focus on relevant areas, the better the model's performance. Considering the different designs of image neural network architectures, the efficacy of each image feature extraction model is strongly associated with the characteristics of the training dataset employed. The transformer-based image feature extraction neural structure, Swin V2, thrives on extensive datasets, as evidenced by the Late model's more comprehensive image data, which significantly enhances its ability to identify relevant plant features, thus allowing for a broader and more accurate activation area in the Late model's Swin V2.

### 4.3 Comparative model design comparison

In comparative studies between one-shot and five-shot learning, Transformer-based (TF) models consistently demonstrated superior performance over Fully Connected (FC) models in the mid and late testing datasets. As highlighted in Fig. 6, TF models achieved the highest scores in 14 instances for one-shot learning, compared to only 10 for FC models. Similarly, in the context of five-shot learning, TF models led in 17 instances against 7 for FC models (Fig. 7). However, FC models exhibited a distinct advantage in the testing result from the Early dataset. The strengths of FC models in the Mid and Late datasets are primarily concentrated at the earliest and latest recorded time points of the flowering period. Specifically, these include 16 days before flowering in the Mid dataset and 12 days before flowering in the Late dataset for the earliest recorded points, as well as 8 days before anthesis for both the Mid and Late datasets at the latest recorded points. This variation should be attributed to differences among the datasets, as influenced by weather conditions, resulting in varying durations from a specific growth stage to anthesis in each wheat dataset. Firstly, the growth phase of the Early dataset spans from Z47 to Z59, corresponding to 23 days–7 days before flowering, during which the visual features of the plants were relatively distinctive at each day. In contrast, the time intervals of the growth stages in the Mid and Late datasets are shorter. Therefore, in the Mid and Late datasets, changes in the plant head occurred more rapidly, resulting in shorter image collection windows. However, since data collection was conducted only once daily, the sampling points were insufficient to represent the detailed growth stages of plants in the Mid and Late dataset periods. Wheat from 10 to 14 days before anthesis undergoes significant visual changes without explicit markers, merely categorized roughly as “Ear quarter emerged,” “Ear half emerged,” and “Ear three quarters emerged.” However, predictions made 16 and 8 days before anthesis have distinct growth stage markers, namely “Flag leaf sheath opening” and “Ear emergence complete,” which provide clear image recognition capabilities. Moreover, through comparative analysis of models with and without weather data (Fig. 9), it was observed that weather data became critical when image information was insufficient, or features were relatively unclear. Therefore, we can conclude that when the representativeness of plant image information is insufficient, necessitating the support of weather data for predictions, TF models demonstrated an advantage.

TF models have an advantage because they are capable of using attention mechanisms to measure the importance of patches through their interactions [36,51]. This capability makes it possible to capture several long-range dependencies and highlight crucial interactions between distant image and weather data feature [52]. In agricultural phenotyping, especially when predicting flowering times, features extracted from environment and imagery phenotypic data are inherently sparse. TF models, which are architected with Multi-Head Attention mechanisms, adeptly discern critical interdependencies among discrete, infrequently occurring features, thereby substantially augmenting the efficacy of the prediction process [53,54]. In summary, FC models perform well in scenarios with clear distinctions in image features, such as predictions from early datasets and when plants are at the earliest or latest recorded time points. On the other hand, TF models are particularly suited for tasks that require detailed integration of spatial and temporal data. However, the complex structure of TF models can increase computational time and reduce performance efficiency when image data alone provides clear information.

### 4.4 Three-class classification

According to the results shown in Fig. 9, the model successfully leveraged previously collected data from similar environments to establish prediction anchors. In the binary classification task, these reused anchors achieved comparable prediction performance to those built from the target dataset itself, demonstrating strong transferability under similar environmental conditions. Although the model performance exceeded 0.6, proving its feasibility to some extent, the three-class classification did not achieve the agricultural application acceptance accuracy of 0.75 (Fig. 9). Moreover, simply expanding the dataset does not guarantee improved model performance. A significant challenge likely stemmed from the method of data collection. Wheat flowering times are concentrated primarily around two periods, morning and evening [46]. Due to the limitation of labour, this experiment only recorded one time point per day, leading to some inaccurate actual flowering time point entries. The flowering time point was crucial, as flowering in the evening indicated that the plant had completed its flowering process after absorbing the day's thermal energy [55]. However, because the recording time point was on the following day, the dataset erroneously indicated an extra day of thermal energy absorption for that plant. In addition, flowering times are often highly sensitive to short-term weather fluctuations, particularly during the final few days before anthesis. Sudden spikes in temperature or solar radiation in the last couple of days can accelerate development, causing plants to flower earlier than expected [56,57]. This biological volatility introduces greater uncertainty into narrow classification windows, especially when attempting to distinguish whether anthesis occurs exactly one day before, on, or after the critical period. In summary, the model did not meet the agricultural accuracy standard of 0.75, largely due to inadequate data collection methods that recorded only one time point per day, coupled with the inherent difficulty of predicting flowering within narrow temporal margins under fluctuating field conditions, affecting the accuracy of crucial flowering times. This highlights the necessity for more precise data capture and dynamic environmental tracking to enhance model reliability in agricultural applications.

In the context of wheat breeding, the shift from a binary to a three-class model that refines flowering time predictions to a two-day window represents a significant advancement. This model not only offers more precise predictions but also improves the timing for critical tasks such as pollen extraction, essential for generating double haploids in hybrid breeding programs. In wheat breeding, anther culture is a critical technique used to produce double haploids, facilitating the creation of homozygous lines essential for subsequent hybrid breeding [58]. The timing of anther extraction is a pivotal aspect of this process. Anthers must be harvested before flowering to ensure the viability of the pollen, which quickly deteriorates once exposed to air [59]. The optimal time for anther extraction in wheat is approximately 4–7 days before anthesis [58]. In hybrid breeding, emasculation should be completed approximately five days before flowering to facilitate cross-pollination, as performing this task too early or too late can significantly impact the success of subsequent crosses [60]. Thus, the three-class model's ability to more accurately predict the pre-flowering period is instrumental. This enhanced predictive capability allows breeders to efficiently manage large-scale wheat fields, scheduling the extraction of pollen and emasculation of wheat plants with greater precision.

## 5 Conclusion

In this study, we demonstrated how innovative multimodal models, incorporating a fusion of RGB imagery and meteorological data combined with few-shot learning approaches, enable the prediction of anthesis in individual wheat plants. Our models proficiently predict whether a wheat plant will flower before or after a predefined critical day, a tool that is extremely useful for wheat breeders and GM field management. The results confirmed that F1 scores approached 0.8 across different planting regimes, indicating a satisfying level of prediction accuracy. The integration of image and weather data significantly improved model accuracy, particularly for the 12–16 days before flowering when the wheat heads were still developing. Few-shot learning allows the models, once trained on one dataset, to be applied flexibly to different datasets, thereby reducing the need for repeatedly collecting large amounts of data and enhancing the models' adaptability and broad applications. This dynamic solution addresses the challenge of predicting the flowering period of individual wheat plants in the field through the use of a novel multimodal approach, significantly improving the adaptability of our models with few-shot learning. Beyond wheat, the multimodal few-shot learning framework developed in this study offers potential applications for flowering prediction in other crops where anthesis timing is agronomically significant. For example, in rice (Oryza sativa), accurate prediction of flowering time is essential for optimizing yield potential, synchronizing flowering in hybrid seed production, and managing abiotic stress during reproductive development. Rice phenology is also highly sensitive to environmental fluctuations, such as temperature and photoperiod, making it a suitable candidate for similar multimodal approaches. By leveraging both image-based cues and weather variables, the proposed framework could be adapted to support field-level flowering monitoring in rice and potentially reduce dependence on labour-intensive manual scoring.

One of the limitations of our approach is that the models can only predict whether the cultivated plant will flower after or before *n* days, without being able to accurately predict the actual day of flowering. The models are currently only applicable to wheat in the Z47 to Z59 growth stages, when the wheat plants have developed part of the head for accurate prediction. Furthermore, although the dataset included three sowing dates, two growing environments, and an independent external test set, all data were collected within a single year (2023). This temporal limitation may restrict the model's ability to generalise to inter-annual variations in climate and plant development. To further improve robustness and applicability, future studies should incorporate multi-year datasets across diverse seasonal conditions. In terms of further work, our focus was primarily on spring wheat varieties in this study, which are cultivated under specific environmental conditions that differ markedly from those of winter wheat. Winter wheat undergoes vernalization—a crucial growth phase involving exposure to low temperatures, typically below 0 ​°C, which is essential for the individual plant's flowering and subsequent development. This difference in growth conditions between spring and winter wheat suggests a potential area for further research, particularly in adapting the multimodal and few-shot learning models to accommodate the unique phenological needs of winter wheat. Moreover, while our method has been proven effective for predicting the approximate flowering times around critical periods essential for wheat breeding, achieving actual flowering time point prediction remains a challenge. Future improvements could focus on refining the recording methods for flowering times and enhancing the accuracy of GDD calculations. Precise GDD tracking is vital as it directly influences the developmental stages of wheat, and improving this aspect could lead to more accurate and actual flowering time point predictions.

## Author contributions

Yiting Xie: Methodology, Investigation, data analysis, Conceptualization, Writing manuscript; Stuart J.Roy: Conceptualization, Methodology, Data interpretation, Writing manuscript; Rhiannon K. Schilling: Methodology, Data interpretation, Writing manuscript; Huajian Liu: Conceptualization, Methodology, Data analysis, Writing manuscript.

## Data and model weight availability

https://github.com/EtingX/Multi-Modal-Few-Shot-Learning-for-Anthesis-Prediction-of-Individual-Wheat-Plants.

## Funding

This work was supported by the ARC Training Centre for Accelerated Future Crops Development IC210100047), 10.13039/501100006670South Australian Research and Development Institute and the University of Adelaide Research Scholarships.

## Declaration of competing interest

The authors declare the following financial interests/personal relationships which may be considered as potential competing interests: Yiting Xie reports financial support, article publishing charges, equipment, drugs, or supplies, and travel were provided by Australian Research Council. Yiting Xie reports travel was provided by International Plant Phenotyping Network. Stuart Roy reports financial support was provided by Australian Research Council. Rhiannon Schilling reports financial support was provided by Australian Research Council. Huajian Liu reports financial support was provided by National Collaborative Research Infrastructure Strategy. If there are other authors, they declare that they have no known competing financial interests or personal relationships that could have appeared to influence the work reported in this paper.
