# Supplementary material for: Multi-modal few-shot learning for anthesis prediction of individual wheat plants
Source: Plant Phenomics. 2025 Jul 21;7(3):100091. doi: 10.1016/j.plaphe.2025.100091 (PMC12709996; doi:10.1016/j.plaphe.2025.100091)
Supplement: Multimedia component 1 [file mmc1.docx]

# Supplementary materials

| 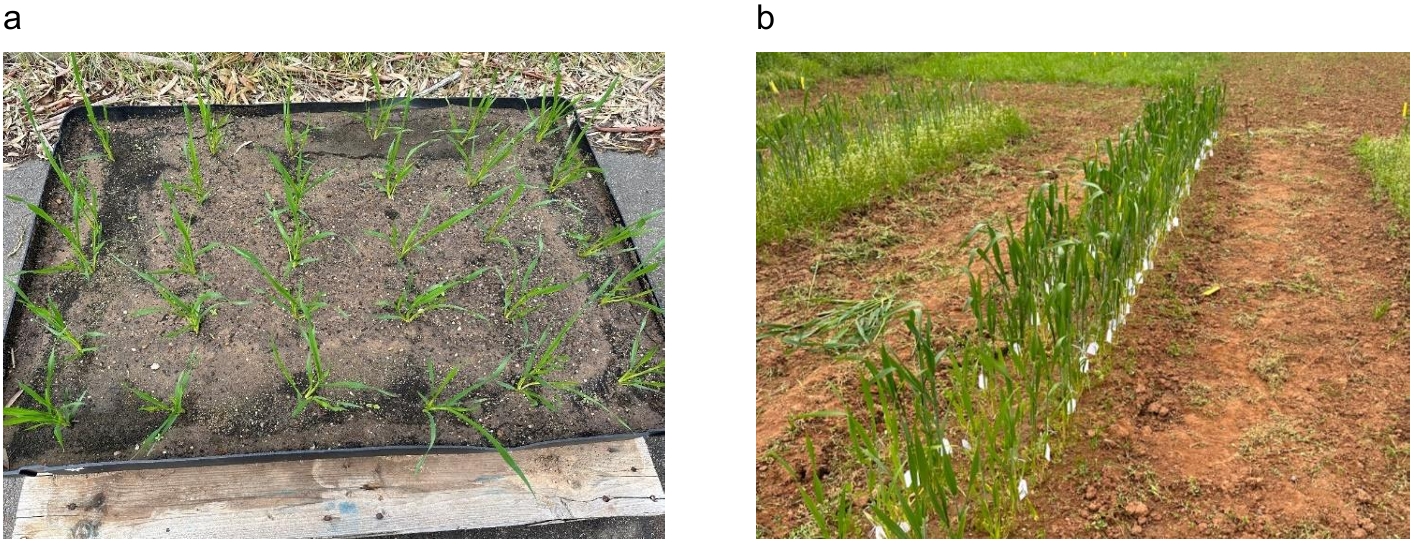 |
| --- |
| Figure S1. Outdoor semi-natural and natural field wheat planting. Figure S1a shows wheat seedlings in large trays, measuring 100 cm by 90 cm by 45 cm, arranged in 5 rows with 6 plants per row, at the Plant Accelerator facility. Figure S1b shows wheat plants in a 10 m length row, adhering to a row spacing of 15 cm and an inter-plant gap of eight to ten centimetres. |

| 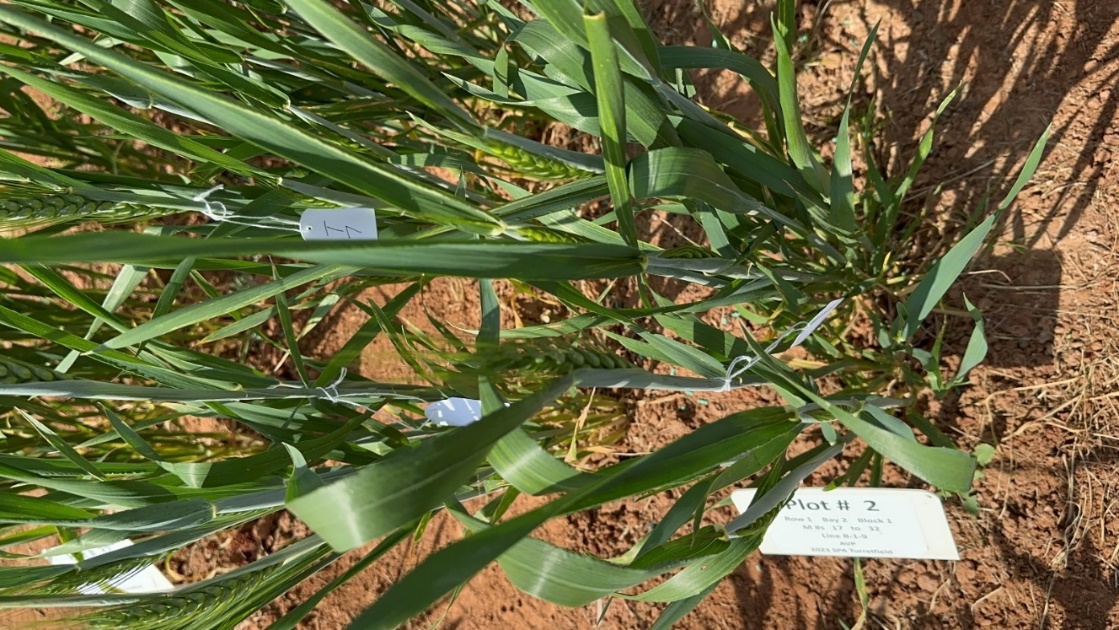 |
| --- |

Figure S2. Individual wheat plant labelling showing the main stem manual tagged to track growth stage and commencement of anthesis.

| 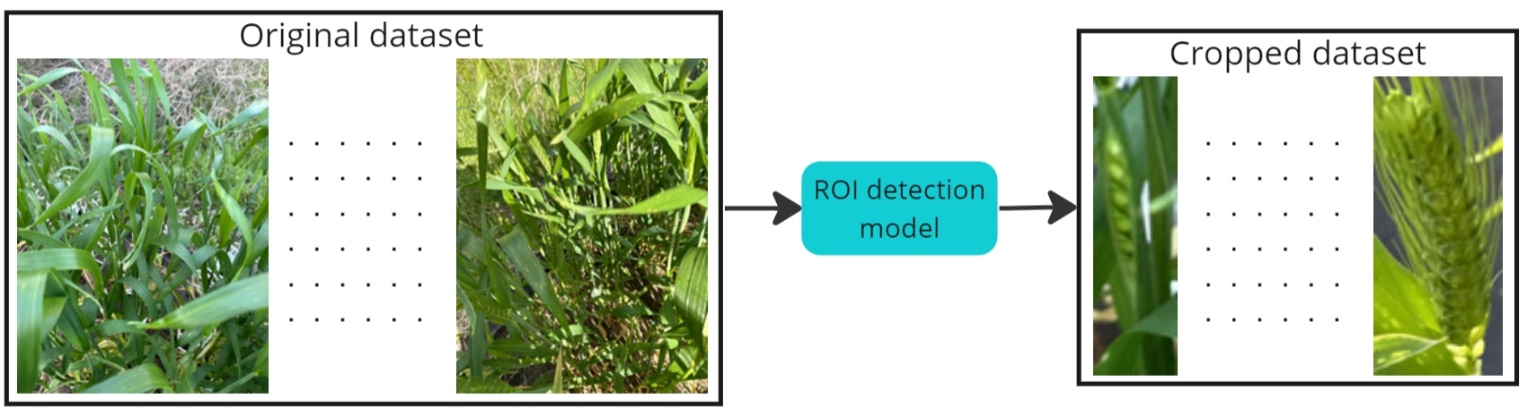 |
| --- |
| Figure S3. Workflow of region of interest (ROI) cropped from wheat image dataset showing the transition from the original wheat field images to cropped images focusing on wheat heads. |

Table S1. Characteristics of wheat datasets across three times of sowing and the dataset characteristics under each condition for the growing condition, number of plants imaged, the range of days before anthesis when images were captured, and the total image count per dataset.

| Dataset | Early (April 11, 2023) | Mid(April 27, 2023) | Late(May 21, 2023) |
| --- | --- | --- | --- |
| Growing condition | Semi-natural conditions (tray) | Semi-natural conditions(tray) | Field natural condition |
| Imaged plants number | 53 | 57 | 103 |
| Days before anthesis | 23 days to 7 days | 18 days to 2 days | 14 days to 5 days |
| Total image number | 6739 | 8255 | 9998 |

Table S2. Key training parameters. Shows hardware specifications, early stopping criteria, and learning rate scheduling strategy.

|  | Parameter |
| --- | --- |
| Device | A100 40GB GPU (Google Colab) |
| Early stop | 3 epoch |
| Cosine annealing | 0.001 to 0.0001 in every 20 epoch |
| Adam optimiser | weight decay=0.0004, Weight decay for bias and batch normalization parameters = 0. |

Table S3. Tukey's honestly significant difference test results for days to flowering from Z47 and Z59 growth stage, respectively.

| group1 | group2 | mean difference | p-adj | lower | upper | reject |
| --- | --- | --- | --- | --- | --- | --- |
| Days from Z47 to flowering | | | | | | |
| Early (Semi-natural) | Late (Natural field) | -7.0491 | 0 | -7.4864 | -6.6118 | TRUE |
| Early (Semi-natural) | Mid (Semi-natural) | -2.9953 | 0 | -3.4866 | -2.5039 | TRUE |
| Late (Natural field) | Mid (Semi-natural) | 4.0538 | 0 | 3.6294 | 4.4783 | TRUE |
| Days from Z59 to flowering | | | | | | |
| Early (Semi-natural) | Late (Natural field) | -5.9749 | 0 | -6.5517 | -5.398 | TRUE |
| Early (Semi-natural) | Mid (Semi-natural) | -4.3198 | 0 | -4.968 | -3.6717 | TRUE |
| Late (Natural field) | Mid (Semi-natural) | 1.655 | 0 | 1.0951 | 2.2149 | TRUE |
